# New Aluminum Alloys Specifically Designed for Laser Powder Bed Fusion: A Review

**DOI:** 10.3390/ma12071007

**Published:** 2019-03-27

**Authors:** Alberta Aversa, Giulio Marchese, Abdollah Saboori, Emilio Bassini, Diego Manfredi, Sara Biamino, Daniele Ugues, Paolo Fino, Mariangela Lombardi

**Affiliations:** 1 Department of Applied Science and Technology, Politecnico di Torino, Corso Duca degli Abruzzi 24, 10129 Torino, Italy; giulio.marchese@polito.it (G.M.); abdollah.saboori@polito.it (A.S.); emilio.bassini@polito.it (E.B.); sara.biamino@polito.it (S.B.); daniele.ugues@polito.it (D.U.); paolo.fino@polito.it (P.F.); mariangela.lombardi@polito.it (M.L.); 2 Center for Sustainable Future Technologies CSFT@PoliTo, Istituto Italiano di Tecnologia, Via Livorno 60, 10144 Torino, Italy; diego.manfredi@iit.it

**Keywords:** laser powder bed fusion, additive manufacturing, aluminum, composition, mechanical properties

## Abstract

Aluminum alloys are key materials in additive manufacturing (AM) technologies thanks to their low density that, coupled with the possibility to create complex geometries of these innovative processes, can be exploited for several applications in aerospace and automotive fields. The AM process of these alloys had to face many challenges because, due to their low laser absorption, high thermal conductivity and reduced powder flowability, they are characterized by poor processability. Nowadays mainly Al-Si alloys are processed, however, in recent years many efforts have been carried out in developing new compositions specifically designed for laser based powder bed AM processes. This paper reviews the state of the art of the aluminum alloys used in the laser powder bed fusion process, together with the microstructural and mechanical characterizations.

## 1. Introduction

**Figure 1:**
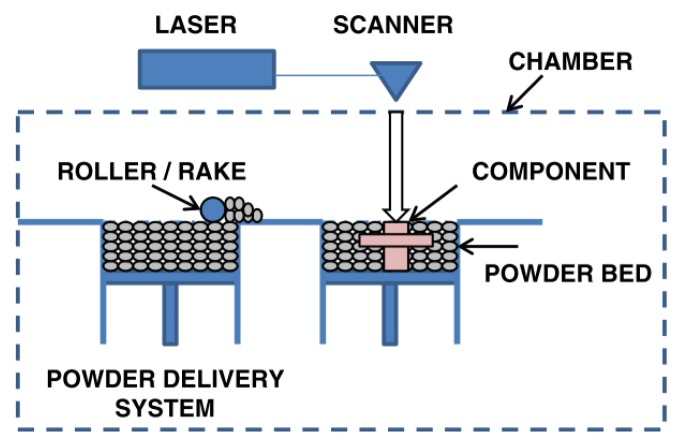
Schematic representation of a laser powder bed fusion (LPBF) process. Adapted from [1], with permission from © 2014 Springer Nature.

Additive manufacturing (AM) defines a class of production technologies that can create 3D components layer by layer based on 2D patterns defined by slicing the 3D computer aided design (CAD) of the component. Among the different AM technologies, powder bed technologies such as laser powder bed fusion (LPBF) and electron beam melting (EBM) are the most used to process metallic alloys [1]. A schematic representation of the LPBF process is reported in Figure 1.

The advantages from a design perspective of LPBF and in general of AM processes have been widely addressed in recent years [2,3]. However, it is important to underline that AM brings many interesting aspects also from a material science perspective. The high cooling rate (10^3^–10^6^ K/s [4]) of the melt pool generated by the successive laser scan tracks is also a key factor of the success of these technologies. From a metallurgical point of view in fact, the rapid cooling generates the solidification of peculiar microstructures characterized by extremely interesting features which will be discussed more in details in the paper. 

LPBF showed to be very successful with many alloys such as titanium, aluminum, nickel, steels, and refractory materials [1]. In the case of titanium and nickel alloys, the poor machinability and the issues related to their formability drove the success of AM processes. On the contrary, most of the aluminum alloys can be easily formed by conventional processes. Furthermore, many studies reported that the LPBF process of Al alloys are generally critical. One of the main issues faced when processing Al by AM is related to difficulties in spreading the powder bed due to the poor flowability of the powders [5]. Furthermore the LPBF process of aluminum alloys generally requires high laser power because of the high reflectivity of the powder and the high thermal conductivity of the solidified material [6]. Finally, the presence of a thin oxide film on the gas atomized particles and on the solidified layers might reduce the processability as it reduces the wettability and hinders the remelting of the previous layer, causing some porosities in the built parts [7,8,9]. The thickness of the oxide layer on both, the melt pool and the particles, strongly depends on the processing conditions [10,11]. Uslan et al. for example showed that on aluminum gas atomized particles the oxide thickness is in the range of 2.75–2.96 nm and it is correlated to the particles size [11]. Notwithstanding these issues, the LPBF processing of Al alloys gained much interest in recent years mainly due to the peculiar microstructure and enhanced mechanical properties it is possible to achieve. 

Among commercial aluminum alloys, mainly near eutectic Al-Si alloys, such as AlSi10Mg, Al-12Si, A357, and A356, are generally used in AM processes and in particular in LPBF and, among them, the most studied composition is certainly AlSi10Mg [12,13,14,15,16]. The success of this composition is mainly related to the Si content, which is close to the eutectic one and hinders the solidification cracking phenomenon. It is well-known that this cracking mechanism is related to the solidification range of the alloy, to the fluidity of the molten phase, the solidification shrinkage and to the coefficient of thermal expansion (CTE) of the alloy [17]. In AlSi10Mg, the presence of 10 wt.% Si implies a fine solidification range (ΔT = T_liquidus_ − T_solidus_) of this alloy, which was calculated to be about 30 °C [18]. This value is extremely low with respect to other high strength aluminum alloys such as the 2024 (ΔT = 135 °C) [19]. In addition, it well-known that Si improves the fluidity of molten aluminum, reduces the solidification shrinkage and the coefficient of thermal expansion [19]. Furthermore, Sercombe et al. suggested that Si is also fundamental for the AlSi10Mg LPBF processing because it is responsible for the laser absorption. Silicon in fact has a low solubility in Al, and it is mainly contained in the alloy as pure particles which are characterized by a high laser absorption (~70%) [20]. 

**Figure 2:**
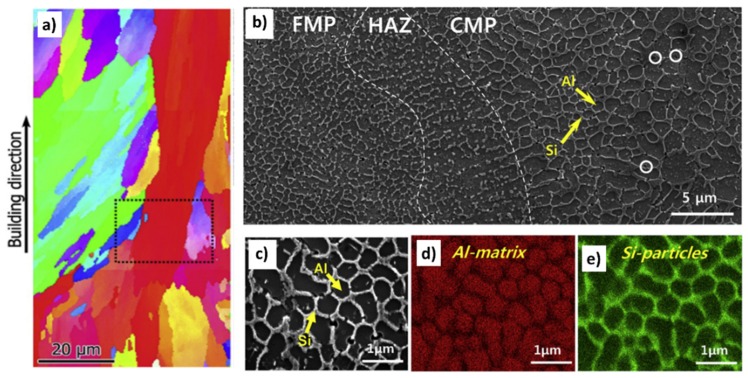
(**a**) Electron backscatter diffraction (EBSD) inverse pole figure (IPF), adapted from [31], with permission from © 2016 Elsevier, (**b**,**c**) SEM images and (**d**) Al- and (**e**) Si EDS maps of an as-built AlSi10Mg sample, adapted from [22], with permission from © 2016 Elsevier.

The microstructure and properties of AlSi10Mg by LPBF have been widely investigated in recent years [12,21]. It is well-accepted that as-built AlSi10Mg LPBF microstructure is made by large columnar grains with 10–20 microns width and hundreds of microns length (Figure 2a). These grains, which can be detected only by electron backscatter diffraction (EBSD) analyses, are formed during the building process thanks to the epitaxial grain growth along the building direction. It is well known that these large columnar grains contain a fine dendritic structure, due to the rapid and directional cooling that arises during the laser scanning and implies enhanced mechanical properties. The effect of the laser scanning can be detected by optical or scanning electron microscope (SEM) images by a network of interconnected melt pools that contain an extremely fine cellular structure. The size and the morphology of the cells strongly depend on the building parameters and on the position within the melt pool [18,22]. Kim et al. for example distinguished three regions of the melt pool defined as, fine melt pool (FMP), heat affected zone (HAZ) and coarse melt pool (CMP) characterized by cells with different size and morphology (Figure 2b) [22]. EDS analyses showed that this cellular structure is made of Al cells surrounded by Si-rich regions (Figure 2c–e).

It was demonstrated that as-built samples are characterized by higher mechanical properties with respect to cast plus conventional solution-ageing T6 treatment samples with the same composition [23]. Hadadzadeh et al. attributed the high mechanical performances of AlSi10Mg LPBF samples to the Hall-Petch mechanism, due to the eutectic cells boundaries, to the Orowan effect due to the presence of Si and Mg_2_Si and to the dislocation hardening [24,25].

Because of this reason, LPBF AlSi10Mg was deeply investigated in terms of post process heat treatments, surface roughness, residual stresses, corrosion and fatigue resistance [26,27,28,29]. 

Despite the results obtained with AlSi10Mg showed the great potentiality of the LPBF process of Al alloys, only other few casting alloys have been processed, without meeting the strict requirements of high strength and ductility of the aerospace industry. In fact, most of the high strength aluminum alloys such as 2000, 6000, and 7000 series are hardly processable by LPBF because of their solidification cracking susceptibility. In addition, 2000, 6000, and 7000 series contain volatile alloying elements such as Zn, Mg, and Li that can easily evaporate during the building process [17,30]. 

**Figure 3:**
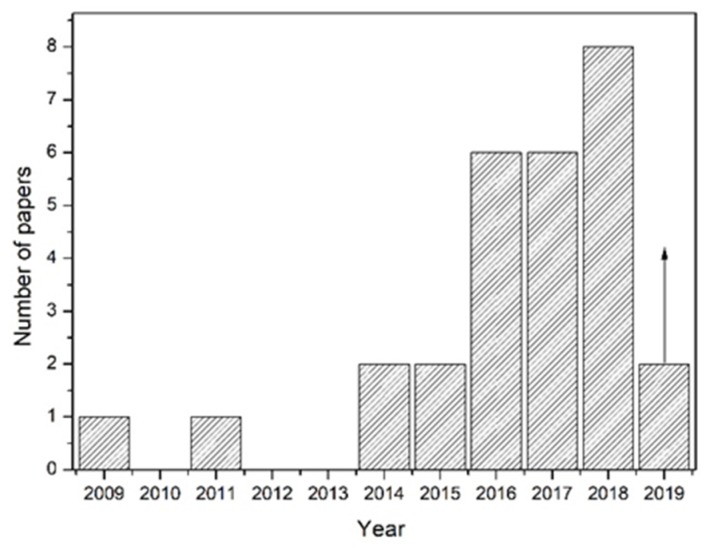
Number of papers published per year on new alloys for LPBF process (author’s image).

Because of these reasons, there is a strong industrial interest in the development of high strength aluminum alloys to be processed by LPBF. Even if the research on this topic started only a few years ago, there has been a significant increase in the number of scientific publications on this topic. Figure 3 reports the number of publications per year on new Al alloys specifically designed for AM processes and clearly demonstrates the increasing interest on this topic. 

The purpose of this review is to discuss the approaches used in the selection of the alloy composition and to sum up the main results obtained in recent years. The major outcomes are here discussed as follows. At first the main methodologies used to study new aluminum alloys compositions are discussed. Afterwards, the advantages of the rapid solidification processes to which LPBF belongs will be presented. Then, the limitations related to the LPBF processability of high strength aluminum alloys (e.g., 7075, 2024) will be presented together with the consequent investigated composition adjustments. Finally, the design of new aluminum alloys obtained by the addition of rare earth (RE) and transition metals (TM) will be examined together with Al-based metallic glasses. As a conclusion the mechanical properties of the alloys will be compared and discussed on the basis of the microstructural results and with emphasis on the key findings.

## 2. Methodologies

**Figure 4:**
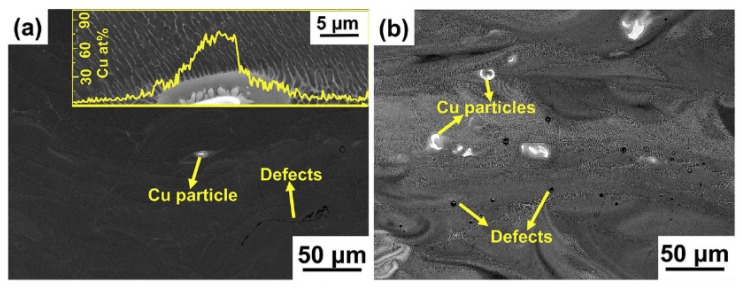
Backscattered Electrons (BSE) SEM micrographs of LBF (**a**) Al-6Cu and (**b**) Al-40Cu alloy (inset: Cu distribution across the Cu-rich zone), adapted from [38], with permission from © 2018 Elsevier.

One of the main issues related to the development of a new aluminum alloy for LPBF process is the difficulty to source gas atomized powders with customized compositions. Furthermore, in some cases, gas atomization of specific compositions might be difficult [32]. Because of these reasons, in recent years many researchers mixed commercial powders with different chemical compositions to obtain a batch with the final required alloying elements content [32,33,34,35,36,37,38]. The phenomena that arise in the melt pool, such as the Marangoni flow and the recoil pressure effects, generally allow the distribution of the alloying elements within the melt pool [39]. Despite this, some of the published studies reported some alloying elements mixing issues [33,38]. Wang et al., for example, produced Al-xCu alloys using mixed Al-4.5Cu and Cu particles and found some Cu rich areas in as-built samples (Figure 4). The inhomogeneity of the composition is strongly undesired not only because it alters the results, but also because it hinders the production of large samples due to the formation of some areas rich in brittle phases.

Furthermore, another critical aspect in developing a new composition is the quantity of powder required for an LPBF build. Accordingly, some researchers used an alternative method to reduce the powder quantity needed to investigate the processability and the properties of a new alloy: laser single scan tracks (SSTs) [40,41,42,43,44]. In these experiments, the laser scans a powder bed with a single track and thereafter the SSTs are generally cut perpendicularly to the scanning direction, polished and analyzed. The morphological observations of the melt pool indicate the processability of the compositions, based on the type melting and solidification phenomena. This method leads to the identification of the most suitable scanning parameters that brings to the formation of a stable melt pool and of a continuous scan track. These types of track are generated when the material melts in conduction melting mode. On the contrary, when high power associated with low scan speed are used the powder bed melts in a keyhole mode, causing a strong evaporation of some alloy elements, the formation of deep melt pools and many undesired effects [45]. The sets of parameters that, because of surface tension effects, cause the rupture of the scan track into separated balls are also undesired as they strongly increase the porosity of the final part. This morphology is generally identified as balling [46].

The main geometrical feature of the cross-section of SSTs can be evaluated in order to select the most suitable hatching distance and layer thickness for the production of dense bulk components. Finally, the melt pool microstructure is generally observed by SEM and EBSD analyses and the mechanical properties are evaluated by nanoindentation measurements [41,42,47]. 

This approach is very useful to have a first insight on the processability, properties and microstructures of a new alloy by LPBF. However, some aspects have to be taken into account:

- the dimension of the laser scan in a real part might be slightly larger with respect to the SSTs due to the heat accumulated during the scanning of the previous layers;
- the microstructure of a SST might be slightly different from the real part one because it does not undergo the intrinsic heat treatment due to the melting and solidification of the following layers.

Notwithstanding this, if these aspects are considered, the SSTs analysis showed to be a promising methodology for the development of new alloys for LPBF. Nie et al. confirmed the applicability of single scan methods by selecting the building parameters for the production of an Al-Cu-Mg alloy on the basis of single and multi-tracks morphology and crack density [41]. 

**Figure 5:**
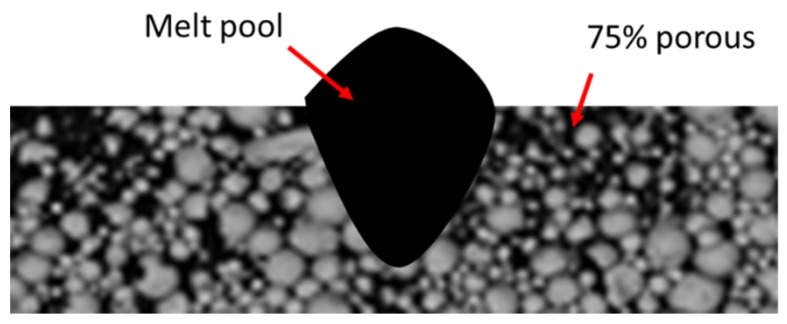
Schematic representation of a melt pool on a partially consolidated powder (This figure was redrawn based on Figure 3 of [42], with permission from © 2014 Elsevier).

In literature there are different methods and approaches to create the powder surface to be scanned. Aversa et al. for example used a film depositor with a 50 μm gap to spread the powders mixed with 50 vol.% ethanol in order to facilitate the deposition [40]. In this case, in order to simulated as much as possible a scan in a real AM part, specific attention has to be payed to the platform composition. Li et al. slightly consolidated a pre-alloyed powder in order to be able to remove the scan track for further analyses (Figure 5) [42]. This approach may facilitate the analyses as it does not involve the platform, but it must be carefully used for the selection of building parameters because the partially consolidated powder bed has a different thermal behavior with respect to the bulk material. Finally, Jia et al. did not create a powder bed but performed SSTs on cast platforms [48]. This method has many advantages from a homogeneity point of view; furthermore critical alloying elements can be added without too many safety issues. However, the lack of the powder bed may modify the laser absorption and the melting phenomena. It is well-known that the laser absorption depends on the surface quality and strongly changes when a powder bed is used [43]. Notwithstanding this, this method is mainly useful for the understanding of the solidification phenomena and microstructure analyses. 

## 3. Rapid Solidification

In recent years many studies modelled the melt pool temperature during LPBF revealing the extraordinary temperature profile and cooling rates of these technologies [49,50,51]. Li et al. for example calculated that the melt pool maximum temperature for an AlSi10Mg is in the range 1000–1800 °C and the cooling rate is in the range 1.2 × 10^6^–6.1 × 10^6^ K/s, depending on the main building parameters of the LPBF system employed [49]. 

These results suggested that LPBF may be considered a rapid solidification process (RSP). As reported in literature, RSPs have been deeply investigated in past years because of their many advantages such as [52]:

- the extension of the solid solubility;
- the formation of non-equilibrium and metastable phases;
- the reduction of number and size of segregated phases;
- the changes in grain morphology such as grain refinement, location and distribution of the phases;
- the reduced phase crystallinity.

The main RSPs are the following: melt spinning (or planar flow casting), splat quenching, laser surface melting and several types of atomization [52]. These technologies produce ribbons or powders and have many limitations in terms of the shape and size of the final products. Nevertheless, because of the possibility to obtain peculiar microstructures and interesting mechanical properties, these processes and their thermodynamic phenomena received much attention in past years in metallurgy literature [53,54]. Many studies in fact focused the attention on the selection of the most suitable composition that can be processed by RSPs and that can take advantage of these extremely high cooling rates [52].

Recently, Marola et al. compared the microstructure of LPBF AlSi10Mg samples with AlSi10Mg specimens produced with common RSPs, in particular melt spinning (MS) and copper mold casting (CMC) [55]. The comparison was carried out by means of Field Emission Scanning Electron Microscopy (FESEM) micrographs and consequent evaluation of the eutectic percentage, X-Ray Diffraction (XRD) and differential scanning calorimetry (DSC) analyses. This study demonstrated that the LPBF samples have a higher extension of the Si solid solubility in Al with respect to the conventional RSPs. This result was verified by the released enthalpy during DSC analyses. These findings confirm that LPBF can be fully considered as a RSP and that it can therefore benefit, from a material point of view, of the above discussed advantages of these techniques. 

## 4. Aluminum Alloys for LPBF

### 4.1. Processability of High Strength Aluminum Alloys

Many studies already revealed that most of the high strength aluminum alloys are hardly processable by LPBF as they suffer from solidification cracking during the laser scanning [17], [30]. This cracking mechanism, deeply investigated in welding, arises when, during the melt pool solidification, the thin liquid film that forms on the grain boundaries cannot accommodate the solidification shrinkage, generating a crack [56]. It was demonstrated that solidification cracking is mainly due to some alloy characteristics, i.e., the large solidification range, the solidification shrinkage, the CTE value, and to the poor fluidity of the molten phase [56]. Furthermore, high strength aluminum alloys usually contain volatile alloying elements such as Zn, Mg, and Li. These elements can evaporate during the LPBF process causing a modification of the composition and consequently of the microstructure and the properties. In some cases, the modification of the composition due to the evaporation might even increase the susceptibility to the cracking mechanism [30].

The occurrence of the cracking mechanism during LPBF of high strength aluminum alloys was demonstrated in several studies [57,58,59]. The processability of an Al-Cu-Mg alloy (with a composition close to 2024) has been investigated by Zhang et al. who observed long cracks formed along the building direction with most of the parameters [57].

**Figure 6:**
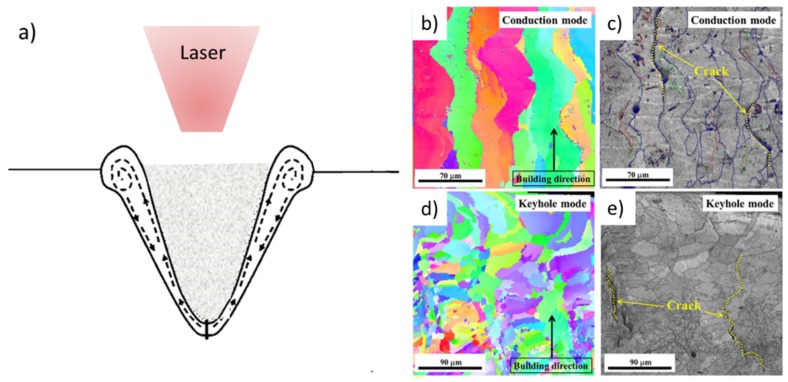
(**a**) Schematic representation of a keyhole melting and EBSD maps of 7050 samples produced in (**b**,**c**) a conduction melting mode and (**d**,**e**) a keyhole melting mode. (**b**,**d**) orientation image map and (**c**,**e**) grain boundary misorientation angle map. Adapted from [59], with permission from © 2017 Elsevier.

Kaufmann et al. studied the LPBF process of the 7075, an aluminum alloy known thanks to its outstanding strength [58,60]. It was demonstrated that with high power (P > 300 W) nearly dense samples (porosity < 1%) can be obtained using a 7075 powder. However, the high cooling rate causes the generation of long cracks, oriented along the building direction also on samples built on a preheated platform (T = 200 °C) [58]. Furthermore, in this study energy-dispersive X-ray spectroscopy (EDS) analyses revealed that LPBF samples contain a lower Zn content with respect to the starting powder, confirming that Zn evaporates during the building process. Qi et al. deeply studied the cracking phenomena that arise during the laser scanning of a 7075 powder [59]. They proved that three different types of cracks could generate in 7075 LPBF samples, all oriented along the grain boundaries. The melting mode was also evaluated on the basis of the geometrical features of the last layers melt pools and was correlated with the crack density. The lowest crack density was observed in samples in which the keyhole melting mode (Figure 6a) arose due to the solidification of fine and irregular grains [45,59]. With high energy density, therefore with high laser power and low scan speed, the keyhole phenomenon takes place due to the evaporation of metal and the consequent creation of a deep melt pool which enhances the laser absorption (Figure 6a) [45]. The EBSD maps of samples built in conduction mode (Figure 6b,c) show that cracks are present along the columnar grain boundaries. On the contrary, the keyhole melting mode causes the formation of irregular grains due to the strong the fluctuation of the melt pool. The different microstructure causes a reduction in the crack density (Figure 6d,e).

### 4.2. High Strength Aluminum Alloys Modification

Considering the difficulties in LPBF processing high strength aluminum alloys, many studies tried therefore to modify their composition in order to make it processable by AM [4,32,34,36,37,41,57]. 

Montero Sistiaga et al. and Aversa et al. modified the composition of the 7075 alloy increasing the Si content, trying in this way to change the solidification range and the fluidity of the molten phase [36,37]. In both cases, the introduction of Si has been obtained by dry mixing the 7075 powders to Si or to a Si-rich aluminum alloy powder. It was demonstrated that Si is very effective in reducing the cracks density of the alloy. DSC analyses confirmed the reduction of the solidification range and EBSD analyses performed on samples with different Si contents showed that silicon also causes a strong grain refinement. This microstructure refinement might also contribute to the improved processability of this alloy. Based on Martin et al. [32] and Qi et al. [59] results, cracks propagated along columnar grains. However, the effect of Si on the laser absorption has also to be taken into account. In fact, the alloy with higher Si content might melt in a keyhole melting mode with a consequent grain refinement [59]. The microstructure of these Si modified 7075 alloys is similar to the AlSi10Mg one, consisting of fine α-Al cells surrounded by the eutectic phase. Thanks to the high cooling rates, the as-built samples are constituted by an α-Al solid solution, whereas a direct ageing heat treatment allows the precipitation of strengthening phases. The tensile tests performed on Si modified samples showed that the as-built alloy has the yield and ultimate tensile strengths of 315 and 387 MPa, respectively, and these values can increase up to 11% and 7%, respectively, after the appropriate ageing treatment [37]. 

On the other hand, Martin et al. studied a new approach, based on the refinement of the microstructure, to produced crack-free 7075 and 6061 parts by LPBF [32]. The authors introduced hydrogen stabilized Zr nanoparticles to the powder batches. Zr reacts with Al forming Al_3_Zr fine particles which act as nucleant for the aluminum alloy. The microstructure of Zr modified alloy consists then in equiaxed grains that can easily accommodate the solidification shrinkage allowing the production of crack-free samples. A similar approach was used by Zhou et al. who added 1wt.% Sc + Zr to an Al-6Zn-2Mg alloy [61]. This composition showed to have a good processability and promising mechanical properties which can be further increased by a T6 heat treatment. 

**Figure 7:**
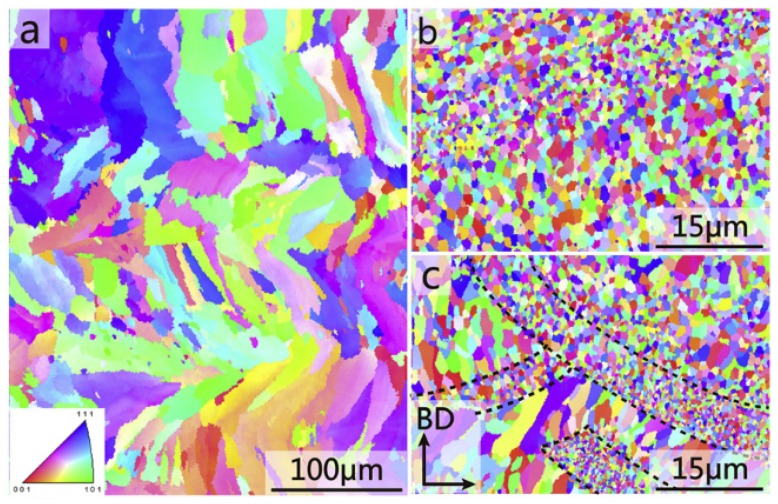
EBSD inverse pole figure (IPF) maps of Al-Cu-Mg fabricated at v = 5 m/min (**a**) and Zr/Al-Cu-Mg sample fabricated at v = 5 m/min (**b**) and v = 15 m/min (**c**), respectively. Adapted from [34], with permission from © 2017 Elsevier.

Similar approaches were used for the alloys belonging to the 2000 series. Dense and crack-free Al-Cu-Mg samples were produced by Zhang et al. only with a reduced range of parameters; with high scan speed, long cracks, oriented along the building direction, were observed [57]. In more recent studies, two main ways to enlarge the process window of this alloy have been studied [4,34,35]. On one hand, Zhang et al. added Zr to its composition in order to form Al_3_Zr precipitates which act as seeds for the heterogeneous nucleation [34]. The experimental results showed that the introduction of Zr is effective in the production of crack-free sample when high scan speed is used [34]. XRD and EBSD analyses confirmed the precipitation of Al_3_Zr which act as a grain refiner for the alloy, allowing to reach extremely high mechanical properties. The EBSD maps of the cross section of Al-Cu-Mg and Zr/Al-Cu-Mg samples processed using different scan speeds are reported in Figure 7. The comparison of the images clearly shows the reduction of the grain size obtained by the introduction of Zr and by using high scan speed values. The reason for the success of the introduction of Zr to the alloy was also clarified by Nie et al. on a similar composition, for which a reduced process windows could be found (Al-4.24Cu-1.97Mg-0.56Mn) [35]. The authors investigated the effect of different Zr contents and different scan speeds on the crack density, revealing that higher scan speed can be used with higher Zr contents [41]. The solidification phenomena have been deeply investigated by EBSD and simulations, confirming that Zr allows the crack reduction thanks to the reduction of the solidification range and the refinement of the microstructure due to heterogeneous nucleation during solidification. 

In another recent study, Wang et al. investigated a different approach to increase the processability of Al-Cu-Mg alloys investigating the effect of the introduction of Si on the consolidation and the microstructure of the starting material [4]. This modification of the composition might reduce the crack density due to the increase in the fluidity of the molten phase and to the reduction in the liquation cracking phenomena. The authors demonstrated that this new Al-3.5Cu-1.5Mg-1Si can be successfully processed by LPBF and that dense and crack-free samples can be produced with the optimized parameters. Concerning the microstructure, it was demonstrated that as-built samples are made of α-Al and strengthening phases and EBSD analyses showed again that large columnar grains are formed upon cooling. XRD and TEM analyses revealed that, in the as-built condition, the strengthening phase is an unknown group identified as Q phase and rich in Cu, Mg, and Si. After the T6 heat treatment, only Mg_2_Si and Al_x_Mn_y_ were detected. 

Finally, the LPBF processability of the alloys belonging to the 6000 series was also investigated and results revealed that these compositions are also very critical [62,63,64]. Louvis et al. reported a poor consolidation and large delamination issues in 6061 samples processed by laser-based AM processes [65]. Fulcher et al. and Maamoun et al. compared the 6061 and the AlSi10Mg processability showing that, because of the larger solidification range, the 6061 alloy suffers from solidification cracking during LPBF [63,66]. In their studies, it was also demonstrated that the cracking mechanism was related to the building parameters and thanks to the rapid cooling, the as-built 6061 microstructure is characterized by fine Al cells and nanometric Si particles [63]. The processability of the 6061 alloy was firstly improved by Martin et al. following the same approach used for the 7075 alloy [32]. The authors introduced Zr nanoparticles in order to cause the precipitation of Al_3_Zr and control the solidification process favoring the heterogeneous nucleation [32]. The 6061 LPBF cracking issues were also recently overcome by Uddin et al. by increasing the building platform temperature at 500 °C and selecting the most suitable building parameters [62]. However, the building platform heating completely altered the solidification mechanism eliminating the melt pool features and leading to a reduced texture, larger grains and precipitates. 

All these studies showed that the three main approaches have been studied for the reduction of the crack density of high strength aluminum alloys processed by LPBF. The methods, based on different microstructural mechanisms, are summarized here:

- Control of the solidification process by the formation of nucleant phases (e.g., Al_3_Zr);
- Modification of the solidification processes by the reduction of the solidification range;
- Reduction of the thermal gradient by preheating the building platform.

### 4.3. Effect of Transition Metals and Rare Earth Elements to Aluminum Alloys

A different approach for the development of new Al-based compositions can be defined on the basis of the results obtained with rapid solidified alloys, introducing transition metals (TM) and rare earth elements (RE) to the alloys compositions [67,68,69]. 

It is well known that TM and RE elements are extremely suitable alloying elements for rapidly solidified Al alloys. Because of this reason, recently, many works focused on the investigation of the microstructure and the properties of aluminum alloys containing RE and TM processed by LPBF. The main advantages of RE are related to the strongly coherent and stable precipitates they form with Al that allow a grain refinement of the alloy.

The most known application of the approach based on TM and RE addition is certainly the Scalmalloy^®^, a new composition developed and patented by the Airbus Group and deeply investigated in recent years. In 2011 Schmidtke et al. proofed the applicability of an Al-Mg alloy containing Sc and Zr through LPBF process (Al4.5Mg0.7Sc0.4Zr0.5Mn) [70]. 

The main idea behind this alloy is related to the obtainment of a supersaturated solid solution due to the rapid cooling and the precipitation of Al_3_Sc by direct ageing. Scandium was selected because, together with erbium and zirconium, is a promising alloying element for aluminum as it gives L12 Al_3_X ordered structures strongly coherent with the Al matrix [71]. This high coherency reduces the tendency of particles to grow, so that these precipitates are stable up to 350 °C. It is worthy to remind that the most common Mg-, Si-, and Cu-based precipitates are stable only up to about 250 °C. Moreover, thanks to their high coherency with aluminum, Al_3_(Sc,Zr) precipitates act as seed crystals for the Al heterogeneous nucleation implying high degrees of grain refinement [72]. 

**Figure 8:**
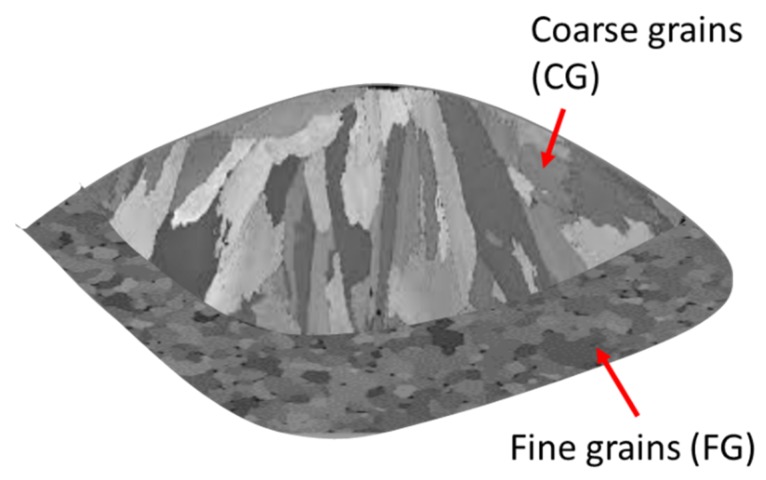
Schematic representation of an Al-Mg-Sc-Zr melt pool microstructure (This figure was drawn based on Figure 2 of [77], with permission from © 2018 Elsevier).

The peculiar microstructure and the properties of Scalmalloy^®^ samples processed by LPBF were investigated in various works by Spierings et al. [72,73,74]. The authors demonstrated that the as-built Scalmalloy^®^ microstructure is made of fine grain (FG) and coarse grain (CG) regions (Figure 8). This bimodal microstructure is probably due to the formation of MgO and Al_3_Sc seeds that cause the formation of fine grains. The CG region, on the contrary, is due to the lack of seeds and to the temperature gradient that cause a columnar grain growth [73]. The authors also investigated the effect of LPBF building parameters on Scalmalloy^®^ microstructure and properties, revealing that the scan speed has an important effect on microstructure and in particular on the size of Al_3_Sc particles. However, this difference only slightly affects the mechanical properties of the as-built alloy in terms of hardness and yield strength [74]. Li et al. also observed the effect of process parameters on the microstructure of as-built LPBF samples with a composition close to the Scalmalloy^®^ (Al-6.2Mg-0.36Sc-0.09Zr). The authors demonstrated that the building parameters have an effect on the texture and on the Mg content of LPBF samples [75]. In particular high energy density caused the evaporation of Mg from the melt pool and a reduction of the Al lattice parameter and the solidification of a structure characterised by a weaker texture [75]. Shi et al. studied the relation between the building parameters and the microstructure and the properties of this composition in the aged condition [76]. It was demonstrated that a balance between the low densification obtained at low energy density and the low supersaturated solid solution obtained at high energy density has to be found in order to reach the highest mechanical performances [76]. 

A similar bimodal microstructure was observed by Griffiths et al. in Sc-free Al-Mg-Zr (Addalloy^TM^) LPBF samples [77]. The authors focused the attention on the effect of laser rescanning on the microstructure of Addalloy^TM^ observing a refinement of the microstructure thanks to the remelting of columnar grains and the precipitation of Al_3_Zr seeds. Croteau et al. also investigated the strengthening mechanisms of Al-Mg-Zr alloys processed by LPBF [78]. The results suggested that the Mg acts as solid solution strengthener while Zr strengthens the alloy based on two main mechanisms, i.e., grain refinement thanks to the precipitation of coherent Al_3_Zr phase during solidification and by precipitation strengthening. 

Because of the extremely promising results obtained with Scalmalloy^®^, various works followed the same idea. Jia et al., for example, investigated the laser processability and the microstructure of Al-Sc-Zr and Al-Er-Zr alloys [48]. Sc and Er form similar L12 precipitates with Al, both extremely coherent with the Al lattice structure [71]. Despite this, the microstructure analyses showed that these compositions have an extremely different behavior: Er has, in fact, a lower solubility in Al with respect to Sc and this causes the reduced thermal stability of these precipitates that easily grow with ageing treatments. Furthermore, EBSD maps confirmed that Er has a different effect with respect to Sc on the solidification mechanisms of Al alloys, implying the solidification of larger grains. 

**Figure 9:**
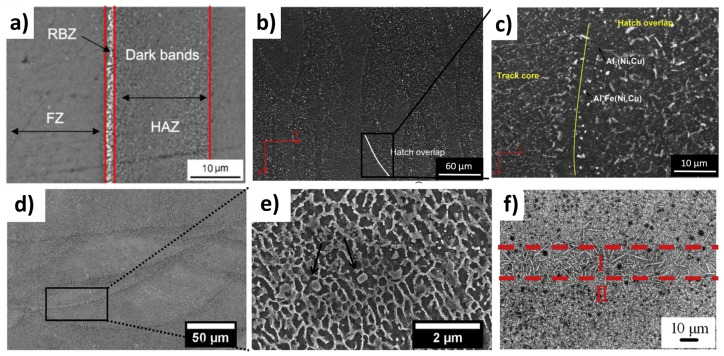
SEM images of (**a**) FVS0812, adapted from [82], with permission from © 2015 Cambridge University Press; (**b**,**c**) Al-Si-Ni-Fe water quenched and aged at 250 °C for 5 h, adapted from [83], with permission from © 2019 Elsevier; (**d**,**e**) Al-Si-Ni, adapted from [33], with permission from © 2017 Elsevier; and (**f**) Al-20Si-Fe-Cu-Mg adapted from [68], with permission from © 2016 Elsevier.

Zheng et al. investigated the microstructure and the properties of the FVS0812 alloy (Al-8.5Fe-1.3V-1.7Si) processed by LPBF [79]. This alloy, developed by Allied Signal Inc., was deeply studied in the 1990s by RSP and powder metallurgy, reaching elevated strengths at high temperature due to the nanoscale Al_12_(Fe,V)_3_Si precipitates [80,81]. Zheng et al. stated that dense and crack-free FVS0812 samples can be successfully produced by LPBF and that as-built samples have a hardness value similar to the planar flow casting one. In the as-built sample microstructure three zones can be recognized: the laser melted zone made of α-Al and fine and round Al_12_(Fe,V)_3_Si, the melt pool border made of fine Al_13_Fe_4_ precipitates and the heat affected zones (Figure 9a) [82]. 

Manca et al. recently proofed the potentiality of a new Al-Si-Ni-Fe alloy containing Cu minor addition, which presented not only a good LPBF processability but also high mechanical performances [83]. The high hardness value of this alloy (186 HV) was attributed to the fine Si, Al_5_Fe (Ni,Cu), and Al_3_(Ni,Cu) phases (Figure 9b,c). 

Aversa et al. studied the processability and the properties of an Al-Si-Ni alloy with a composition close to the ternary eutectic one using AlSi10Mg and Ni particles [33]. From a microstructural point of view, the as-built samples are constituted by α-Al cells surrounded by the eutectic and Al_3_Ni particles. Similarly to what was observed on the FVS0812 alloy, slightly larger precipitates were recognized at the melt pool boundary (Figure 9d,e). The hardness measurements revealed that the introduction of 5 wt.% Ni to the AlSi10Mg composition caused an increase of 23% in Vickers hardness. Nanoindentation measurements were then used to demonstrate that the high hardness value is induced by the presence of fine Al_3_Ni particles. 

On the other hand, Ma et al. studied the effect of Fe, Cu, and Mg addition on the microstructure of an Al-20Si alloy processed by LPBF [68]. It was demonstrated that the microstructure consists of α -Al, Si bulk particles, Al-Si eutectic and coarse acicular δ-Al_4_FeSi_2_ phase (Figure 9f). The size of these phases is strongly lower with respect to the cast ones thanks to the high cooling rate of the LPBF process. Furthermore, the presence of Fe, Cu, and Mg hinders the growth of Si phase. 

Wang et al. investigated the microstructure and the properties of Al-Cu samples with different Cu contents (4.5–40 wt.%) produced by LPBF starting from mixed Al and Cu particles [78]. The microstructures of the low Cu content alloys were made of an Al matrix and Al_2_Cu particles. Moreover, the microstructure became fine eutectic as the Cu content increases. The highest mechanical properties, evaluated by compression tests, were recorded in the case of the compositions closest to the eutectic (Al-33Cu). Despite the sensitivity of Al-Cu alloys to cracking mechanisms, no consolidation issues of these alloys were reported in this paper. 

These studies showed that the success of the Scalmalloy^®^ composition is related not only to the precipitation of L12 coherent strengthening phases but also to the strengthening effect of Mg which is contained in a supersaturated solid solution in the α-Al matrix. Subsequent studies on the effect of other RE elements such as Er demonstrated that the relatively high solubility of Sc in Al is also a key-point for the success of AlMgScZr compositions. 

The introduction of TM to the LPBF alloys compositions can be then considered a promising method to improve the mechanical properties of the alloy by maintaining a good processability. The composition studied up now were mainly based on the most promising alloys processed by RSP. The microstructural and mechanical investigations demonstrated that, due to rapid cooling, fine microstructures and by high mechanical properties can be achieved by LPBF. 

### 4.4. Metallic Glasses and Nanocrystalline Materials

Metallic glasses (MGs) and nanocrystalline materials (NCMs) are two class of materials that received great attention in recent years thanks to their unique properties due to the lack of a conventional metal structure characterized by long-range order [84]. The LPBF processability of a few materials belonging to these class was studied in recent scientific publications. 

Prashanth et al. studied the microstructure and the properties of thermally stable Al_85_Nd_8_Ni_5_Co_2_ nanocrystalline material produced by pre-alloyed gas atomized powder. The authors reported a composite like microstructure made of Ni, Nd and Co-rich sub micrometric platelets like intermetallic precipitates [85]. These particles implied an increase in the mechanical properties of the alloys and a reduction of the microstructure coarsening. Furthermore, the elongated particles, characterized by a strong interface with the matrix, were very effective in deflecting cracks and increasing the mechanical strength. 

Li et al. investigated the possibility to process an Al-based metallic glass (Al_86_Ni_6_Y_4.5_Co_2_La_1.5_) by LPBF [42]. The authors firstly performed SSTs and observed that some process parameters caused cracks within the SSTs. It was also demonstrated that the microstructure and the hardness strongly depend on the position within the melt pool: some areas crystallize due to a different thermal history. In a second study, the same authors investigated the effect of low energy density rescanning in the processing of bulk metallic glass composites (BMGCs) [86]. It has been demonstrated that for these materials rescanning with low power allows the reduction of residual stresses. 

## 5. Mechanical Properties

The main mechanical properties of the above-described alloys in terms of hardness (HV and HB), yield strength (YS), ultimate tensile strength (UTS) and elongation (ε) are reported in Table 1. Undoubtedly, the heat treated Scalmalloy^®^ samples showed the highest YS and UTS values together with a high elongation. The authors attributed this extraordinary mechanical strength to Sc and Zr which form Al_3_Sc and Al_3_(Sc_x_Zr_y_), to Zr that creates a shell around Al_3_Sc particles, and mainly to the presence of Mg which causes a solid solution strengthening effect [70]. It is important to underline that the post processing heat treatment plays a crucial role in determining the properties of the alloy [76], it can be noticed indeed that the as-built Scalmalloy^®^ properties are similar to the as-built AlSi10Mg ones [76]. The comparison of the Scalmalloy^®^ with the Al-Sc-Zr confirms the relevance of both strengthening effects. Furthermore Jia et al. demonstrated that Er does not imply the same strengthening effect of Sc on LPBF Al alloys mainly because of the large size and reduced volume fraction of the strengthening phase [48].

The comparison of the YS values of Al-Cu-Mg-Mn and Zr-Al-Cu-Mg-Mn highlights the positive effect of Zr on the mechanical properties of as-built materials. In this case, the increase in the high mechanical properties was attributed to the grain refinements and to the strengthening effect of the precipitates [34,35]. On the other hand, the increase in the processability obtained by adding Si to the Al-Cu-Mg-Mn composition also allows a slight increase in YS and UTS values, especially after a T6 heat treatment [4]. In this case the strengthening effect was mainly attributed to the Q phase, in the as-built state, and to the Mg_2_Si and Al_x_Mn_y_ precipitation in heat-treated samples [4]. Contrarily, Zr and Si showed to have a similar strengthening effect on the 7075 composition [32,36]. The comparison of the 6061 properties suggests that similar YS and UTS can be obtained by as-built 6061 (T = 200 °C) and T6 6061 built at 500 °C [62,63]. The high mechanical properties obtained by processing materials with lower building platform temperatures are related to the fine microstructure that solidifies thanks to the rapid cooling. However, it must be pointed out that in this case the authors reported the presence of small micro-cracks in as-built samples.

Finally, the strengthening effect of the transition metals can be clearly seen by comparing the Vickers hardness values of AlSi10Mg with the AlSiNi and Al-8.5Fe-1.3V-1.7Si ones. In both cases the high mechanical properties were attributed to the fine Al_3_Ni, Al_12_(Fe,V)_3_Si, and h-Al_13_Fe phases.

## 6. Conclusions

This review aims to demonstrate the strong interest and the great possibilities related to new alloys development specifically designed for AM processes, and in particular Al alloys for LPBF process. It could be stated that, up to now, the several approaches based on the following effects have been used:

1. The reduction of the solidification cracking mechanism of commercial high strength aluminum alloys (e.g., 7075, 2024, and 6061) thanks to the modification of the melting behavior. This effect was obtained mainly by the introduction of Si which increases the fluidity of the molten phase and reduces the alloy melting range, its coefficient of thermal expansion and its solidification shrinkage. The decisive effect of Si was demonstrated on the 7075 and 2024 compositions.
2. The reduction of the solidification cracking obtained thanks to the reduction of the grain size of commercial high strength aluminum alloys achieved as a result of the precipitation of strongly coherent phases. Zr was mainly employed in order to obtain fine coherent Al_3_Zr particles which act as nucleant during the solidification process. This method demonstrated to be promising for the processability of 7075, 2024, and 6061 alloys.
3. The reduction of the solidification cracking by the increase in the building platform temperature. This method implies a reduction of the thermal stresses and therefore of the cracking density. The high temperature of the building platform causes however a reduction in the cooling rate and precludes the solidification of fine microstructures. This method was however successfully applied to the 6061 composition which could achieve high mechanical properties in the T6 condition.
4. The introduction of rare earth and transition metallic elements to standard Al alloys compositions, which implied a strong increase in the mechanical properties of LPBF samples. The most promising composition was undoubtedly produced by the introduction of Sc and Zr to an Al-Mg alloy leading to the patented Scalmalloy^®^ composition. The high mechanical properties of this alloy are mainly due to the precipitation of coherent Al_3_(Sc,Zr) particles and to the Mg solid solution strengthening effect. Furthermore, this composition resulted to be stable up to high temperatures thanks to the Al_3_(ScZr) poor tendency to grow. On the basis of the success of this composition, many authors focused on the study of similar compositions containing Sc, Er, and Zr and obtained promising results. The introduction of less expensive TM elements seems to be also a promising approach for the production of high strength LPBF Al alloys. The rapid solidification achieved during the laser scanning allows the precipitation of extremely fine strengthening phases and therefore high mechanical properties.
5. The production of metallic glass and nanocrystalline materials. Thanks to the rapid solidification that arises as a consequence of the laser scanning, it seems that the LPBF process might be a promising production technology for these materials. However, because of the peculiar thermal history to which the material undergoes, different microstructures can be obtained. This aspect has to be carefully taken into account if complex parts have to be built.

The analyses of the mechanical properties of newly designed alloys showed that it is possible to produce high strength Al alloys by LPBF. The mechanical performances of LPBF Al alloys are mainly due to the fine microstructure and the supersaturated solid solution obtained by the rapid solidification and by the strengthening effect of fine strongly coherent particles. It must also be underlined that, in most of the cases, non-standard heat treatments were used on these materials because of the peculiar microstructure of as-built components.

Furthermore, the results highlight that the microstructure and the properties of LPBF compositions strongly depend on the building parameters used during the AM process. On the basis of this consideration, it is important to underline that a promising composition must be coupled together with the most suitable parameters that allow the solidification of a fine microstructure and a supersaturated solid solution in order to achieve the highest mechanical properties.

The industrial scalability of these compositions also has to be taken into account. The cost of some elements such as Sc, Er, and Zr, used as grain refiners, has to be considered when evaluating the applicability of these compositions. In AM however, unlike conventional processes, the usage of more expensive materials is admitted thanks to a low buy-to-flight ratio close to 1. Furthermore, as demonstrated by the Scalmalloy^®^ example, the high mechanical performances achieved by these compositions can often justify the high cost of the powder. On the other hand, the introduction of silicon is certainly a less expensive modification; however a complete characterization of this composition is necessaire. Finally, when considering the increase in the building platform temperature for the production of crack-free high strength Al alloy parts, the technological limit of this approach has to be considered. Up to now, in fact, most of the commercial systems do not reach sufficiently high temperatures.

To conclude, it must be pointed out that, up to now, data about advanced characterizations, such as fatigue and high temperature properties, on these materials are not available in scientific publications. This knowledge is extremely important to meet the strict requirements of the aerospace industry. This lack of data might be due, on the one hand, to the novelty of these studies and, on the other hand, to the strong industrial interest in this topic which causes to the non-disclosure of specific materials properties. Only advanced mechanical characterizations will allow the definition of the best approach for improving strength and fatigue values of Al alloys for LPBF.

## Figures and Tables

**Table 1:** Mechanical properties of modified aluminum alloys processed through LPBF. Hardness (HV and HB), yield strength (YS), ultimate tensile strength (UTS) and elongation (ε).

Composition	Heat Treatment	HV	HB	YS [MPa]	UTS [MPa]	ε (%)	Reference
AlSi10Mg H		135.0 ± 0.9	128.6 ± 1.9	270 ± 10	460 ± 20	9 ± 2	[33]
AlSi10Mg H	S.R.	~95	93 ± 3	230 ± 15	345 ± 50	12 ± 2	[12]
Scalmalloy^®^	-	-	100–115	276–287	403–427	14–17	[74]
Scalmalloy^®^	325 °C 4 h	~180	-	520	530	14	[70]
AlScZr L.R.	5 h 300 °C	113 HV0.5	-	-	-	-	[48]
AlErZr L.R.	2 h 300 °C	91 HV0.5	-	-	-	-	[48]
AlCuMgMn	-	-		276.2 ± 41	402.4 ± 9.5	6 ± 1.4	[57]
Zr/AlCuMgMn	-	-		446 ± 4.3	451 ± 3.6	2.7 ± 1.1	[34]
Zr/AlCuMgMn	-	153.6		464.06 ± 2	493.30 ± 10	4.76 ± 1	[35]
2219	-	94 ± 6.6					[87]
2219	T6	147 ± 2.3					[87]
Al-3.5Cu-1.5Mg-1Si	-			223 ± 4	366 ± 7	5.3 ± 0.3	[4]
Al-3.5Cu-1.5Mg-1Si	T6			368 ± 6	455 ± 10	6.2 ± 1.8	[4]
7075+Zr	T6	130–140	-	32–373	383–417	3.8–5.4	[32]
Si mod. 7075	6 h 150 °C	~170	-	-	-	-	[36]
Si mod. 7075	6 h 160 °C		140–150	350	415	-	[37]
Al-8.5Fe-1.3V-1.7Si	-	135–175	-	-	-	-	[79]
AlSiNi	-	158.7 ± 3.0	179.5 ± 3.0	-	-	-	[33]
Al-3.60Mg-1.18Zr	400 °C 8 h	-	-	353 ± 5	386 ± 3	18.6 ± 0.9	[78]
Al-3.66Mg-1.57Zr	400 °C 8 h	-	-	365 ± 11	389 ± 4	23.9 ± 4.4	[78]
6061	-	67–84		246.7	392		[63]
6061 500 °C platform	-	54 ± 2.5		66–75	133–141	11–15	[62]
6061 500 °C platform	T6	119 ± 6		282–290	308–318	3.5–5.4	[62]

* H = Horizontal, S.R. Stress Relieved (300 °C 2 h), L.R. Laser remelted.
